# 4363 TCF7L2 variants in African American patients with bipolar disorder and high BMI

**DOI:** 10.1017/cts.2020.186

**Published:** 2020-07-29

**Authors:** Linsey Jackson, Brandon Coombes, Joyce E. Balls-Berry, Joanna Biernacka

**Affiliations:** 1 Mayo Clinic

## Abstract

OBJECTIVES/GOALS: In a previous genome-wide association study of European Americans, rs12772424, a variant in the gene encoding transcription factor 7-like 2 (TCF7L2), was shown to have a SNP-BMI interaction association with bipolar disorder (BD). The objective of this study was to replicate this finding in an African American (AA) sample. METHODS/STUDY POPULATION: Using a sample of 659 controls and 323 BD cases from the Genetic Association Information (GAIN) Study of BD, we conducted analyses to assess association between BD and the interaction of BMI with genetic variation in TCF7L2. For this study we identified 4572 single nucleotide polymorphisms (SNPs) in a 1Mb region around the candidate SNP rs1272424. Based on variants identified in the prior analysis of the larger European American dataset we selected SNPs for analysis in the AA data. This allowed for a smaller AA sample, and still maintained adequate power for statistical significance. RESULTS/ANTICIPATED RESULTS: We anticipate observing an effect of TCF7L2 variation on the relationship between BD and BMI in the AA data. We also anticipate that combining results from the European and African American patients may help narrow down potentially functional variants in TCF7L2 that influence the association between BD and BMI. DISCUSSION/SIGNIFICANCE OF IMPACT: Psychiatric genetics studies lack ancestral diversity among participants, decreasing their generalizability, and possibly increasing health disparities, especially for diseases like BD, which is often misdiagnosed and untreated in AAs. This work propels us towards understanding the genetics of BD and obesity in this underrepresented population.

